# Improvement of Dentin Growth Parameters (Beta-catenin, bFGF, CD105, and BMP4) with Propolis as Adjuvant in Dental Caries Treatment

**DOI:** 10.1055/s-0044-1791939

**Published:** 2024-12-10

**Authors:** Retno Pudji Rahayu, Nirawati Pribadi, Ira Widjiastuti, Nur Ariska Nugrahani

**Affiliations:** 1 Department of Oral and Maxillofacial Pathology, Faculty of Dentistry, Airlangga University, East Java, Indonesia; 2 Department of Conservative Dentistry, Faculty of Dentistry, Airlangga University, East Java, Indonesia; 3 Faculty of Dentistry, Universitas Muhammadiyah Surakarta, Surakarta, Central Java, Indonesia

**Keywords:** beta-catenin, bFGF, CD105, BMP4, propolis

## Abstract

**Objective** The purpose of the study was to evaluate the efficacy of calcium hydroxide (Ca(OH) _2_ ) and propolis in pulp capping for dental caries treatment, focusing on dentin growth parameters. The study also aims to determine the role of propolis as a natural adjuvant therapy in enhancing reparative dentin development while emphasizing the importance of proper technique and material preparation with markers for the expression of beta-catenin, bFGF, CD105, and BMP4.

**Materials and Methods** The left bottom molar teeth from 28 Wistar rats were divided into four groups. The first group, the control group, was given only aqua dest, and the second group received drilling treatment and additional therapies with Ca(OH) _2_ (Ca(OH) _2_ ) 0.625 μg. The third group was given drilling treatment and additional therapies with a combination of propolis with Ca(OH) _2_ 0.781 μg until day 7. Finally, the fourth group received a combination of propolis with Ca(OH) _2_ 0.781 μg until day 14. This research analyzed the expression of essential basic fibroblast growth factor (bFGF), CD105, beta-catenin, and bone morphogenetic protein 4 (BMP4).

**Results** This research reports that the average expression of BMP4 and bFGF showed a significant result in treatment with additional therapies with propolis and Ca(OH) _2_ . The experiment indicates that propolis and Ca(OH) _2_ could induce reparative dentine on days 7 and 14.

**Conclusion** Propolis as an adjuvant shows better reparative dental formation with improvement in the expression of bFGF and BMP4 in 14 days of therapy.

## Introduction

The primary solution for dental caries is pulp capping treatment using calcium hydroxide (Ca(OH) _2_ ) with indirect and direct methods. 1 2 Ca(OH) _2_ can be used for root canal treatment due to its antimicrobial activity and ability to repair complex tissue formation, especially dentin. 3 When Ca(OH) _2_ is applied to the dental pulp, it initiates a vascular response. This response involves cell migration, proliferation, and a process known as liquefaction necrosis (necrotic pulp). These conditions are essential for promoting biomaterial mineralization and cellular differentiation within the pulp. In previous research, Ca(OH) _2_ was frequently used in the field of dentistry. It possesses highly alkaline properties that can lead to inflammation within the pulp. Besides that, Ca(OH) _2_ is indeed a versatile material in dentistry, but its alkalinity can have both beneficial and potentially adverse effects on dental pulp. While it promotes dentin formation and has antimicrobial properties, its extreme alkalinity may cause inflammation if not used judiciously. 4 Bacterial agents can infect necrosis pulp, resulting in continuous tooth adherent cariogenic and inhibition in reparative dentin formation. Therefore, better material may be needed for improvement in therapy outcomes. 4 5

Propolis can efficiently reduce inflammation, bacterial infection, and necrosis, helping form a dense dental layer with stem cell stimulation. 6 7 This simulation can happen because of the flavonoid content in propolis. Research shows that propolis has low toxicity to fibroblast cells and reduces apoptosis, stimulating the proliferation of fibroblast cells. 8 9 With that rationale, we use propolis as an adjuvant in dental treatment with a rat model that has been used many times because it has better mimicry as a human body. 7

The pulp can regenerate to repair dentin in the event of tooth damage. Stimuli such as odontoblast preparation and advanced odontoblast can cause odontoblast death and stimulate odontoblast differentiation of stem progenitor cells population of dental pulp cells (DPCs) that replace necrotizing odontoblast. 10 In addition to other types of cells, dental pulp also contains fibroblasts. Although they do not directly contribute to dentin formation, fibroblasts are vital to the process of extracellular matrix (ECM) production. This process involves the synthesis of collagen and other ECM components by fibroblasts. In addition to their roles in tissue repair and the immune response, fibroblasts also play a role in paracrine signaling by secreting growth factors and cytokines, which can affect nearby cells such as odontoblasts and dental pulp stem cells (DPSCs). For pulp capping and dentin repair to be successful, it is necessary to understand the interplay between odontoblasts, DPSCs, and fibroblasts. Although odontoblasts play a pivotal role in dentinogenesis, the regenerative microenvironment also includes fibroblasts and other cells. 10 11 A combination between propolis and Ca(OH) _2_ will not create a toxic reaction with evidence from previous research and has benefits with the stimulation of reparative dentine. 11 Moreover, propolis has unique flavonoid content, such as caffeic acid phenethyl ester (CAPE), which has multiple beneficial effects.

With that consideration, a plan should be developed to analyze growth parameters, such as beta-catenin, essential basic fibroblast growth factor (bFGF), CD105, and bone morphogenetic protein 4 (BMP4), to determine the beneficial role of propolis in dentin formation. We also investigate which growth parameter characterizes the formation of reparative dental pulp the most for further study and clinical consideration.

## Materials and Methods

### Ethical Permit for Animal Experiment

This study used a posttest-only controlled group design, with a research period from September until December 2021. It obtained an ethical permit for conducting an animal experiment in dental medicine, with an appointment number (20/UN3.9.3/Ethical/PT/2021), which established the proper standards and guidelines that followed throughout this investigation.

### Experimental Study Design

Rats were used as a model for their mimicry of human physiology. From the Charan Biswas formula and dropout factors, we calculated the rats used in this research and found the result was 28. Adult male Wistar rats weighing 250 g at 3 to 4 months of age were used in this research and placed in a cage at a temperature of 27°C with food (Phokphan Hi-Provite) and water provided *ad libitum* in a light- and temperature-controlled environment. To minimize the bias, the researchers randomized the data using a computer software program without controlling the confounding factors. Then, we divided into four groups with seven rats and acclimatized for 1 week. Each group was given the following treatment: Control (no treatment)

1. A1 (positive control), treated with Ca(OH) _2_ .
2. A2 treated with propolis and Ca(OH) _2_ for 7 days.
3. A3 treated with propolis and Ca(OH) _2_ for 14 days.

Dycal from Dentsply, available for pulp capping with a ratio of 1:1, and extract propolis, which is a powdered form of natural raw bee extract from *Apis mellifera* , are among the ingredients, and the concentration of the powder is 0.937 μg. In the dental decay model, the animals were administered an intravenous anesthetic (3% pentobarbital sodium at a dosage of 1 mL/kg) to minimize discomfort. Subsequently, each rat's tooth was carefully drilled with a low-speed 0.8 mm round bur until the pulp chamber was exposed and bleeding occurred. Propolis and Ca(OH) _2_ were applied and mixed until the consistency became a paste. Both control (A1 and A2) group samples were taken on day 7 after induction and therapy. The A3 group sample was taken on day 7, and the A4 group sample was taken on day 14. After that, the composite covers the Ca(OH) _2_ or the propolis/Ca(oH)2 combination on the mouse's tooth. In this study, there should be A1 and A2 controls for the 14th day), but the researcher only provided data in the form of groups A1 and A2, which would be killed on the 7th day only; then, on the 14th day, there would be no more A1 and A2 groups based on evidence-based research. Propolis is the only group not included because the consistency of propolis powder is not the same as the consistency of pasta for pulp capping. Previous research has not yet included only propolis mixed with equates or else for making pasta. 10

#### Outcome Measures

Of all the 28 rats in this study, 3 died from the group (A3, 1 died, and A1, 2 died) during the process on the second day. It showed signs of inflammation in the gingiva and weak immunity so that the rats could not survive at the same time; after observation for 3 days, the rats showed protruding red eyes, did not want to eat and drink, and did not move like other rats. One was excluded from the research because of a lack of willingness to eat. We also examined for inflammation signs in the rat's gingiva. All the rats that still survived and did not show any sickness signs or lethargy had a good gingival condition, and the inflammation signs (redness, swelling, heat, pain, and loss of function) did not occur in the gingival tissue. For sample collection, the rats were anesthetized with ketamine at a dose of 87 mg/kg bb with an injection intramuscular in the right thigh once before sampling.

Human end points established for this study monitored the occurrence of inflammation in mice every day until the end of the study, monitored rats eating every day, and changed drinking water in mice. If mice showed signs of red eyes and lack of activity, they would be separated from the group and transferred to the cage that will be observed (morning, afternoon, evening). Special treatment was given to rats showing pain by adding vitamin C mixed in their drink at a dose of 2.5 mg/300 g; this is our exclusion criterion in the study, but it must still be treated even if it eventually dies.

#### Beta-catenin, bFGF, CD105, and BMP4 Expression with Immunochemistry

Paraffin blocks formed through dehydrating, clarifying, impregnating, and embedding processes were cut using a microtome and placed on the preparation. The histopathological sample was stained by hematoxylin to see macrophages and find the suitable antibody kits to see the expression of beta-catenin (sc-1496 from Santa Cruz Biotechnology, RRID: AB_1249372), bFGF (ab9596 from Abcam), CD105 (MS-1290-R7; NeoMarkers, Inc.; Fremont, California, United States), and BMP4 (ab39973 from Abcam, RRID: AB_2063523).

#### Data Analysis

Data analysis was completed with SPSS (RRID: SCR_002865) version 16.0. The normality test was performed using the Shapiro–Wilk's test, and the homogeneity test was performed using Levene's test. One-way analysis of variance (ANOVA) assessed differences between the groups, followed by the posterior minimum significant difference test used for homogeneous data. A *p* -value > 0.05 was considered significant.

## Results

**Fig. 1:**
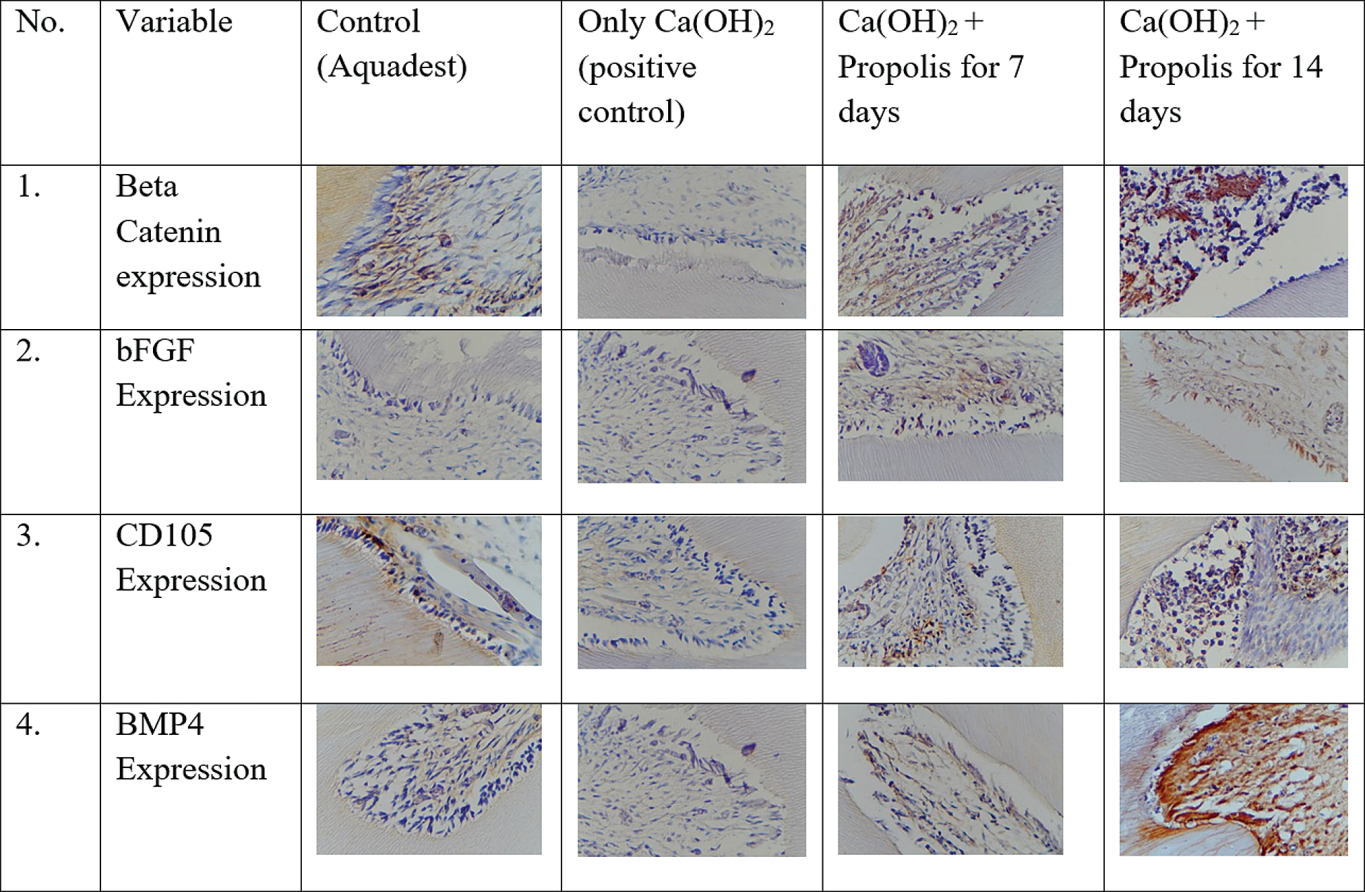
Expression of beta-catenin, bFGF, CD105, and BMP4 from immunochemistry. bFGF, basic fibroblast growth factor; BMP4, bone morphogenetic protein 4.

The mean values of growth parameters (bFGF, CD105, beta-catenin, and BMP4) in the control group treated only with aqua dest were higher than those in a positive control group with Ca(OH) _2_ . The group that combined Ca(OH) _2_ and propolis until day 7 had higher mean values of growth parameters than both control groups. Finally, the group combining Ca(OH) _2_ and propolis until day 14 had the highest mean growth parameter values of all the three groups before ( Fig. 1 ; Table 1 ).

**Table 1:** Expression of bFGF, CD105, beta-catenin, and BMP4 in respective group

No.	Group (mean ± SD)	bFGF	CD105	Beta-catenin	BMP4
1	Control (aqua dest)	5.0 ± 1.89	4.16 ± 1.47	7.33 ± 1.63	6.0 ± 2.19
2	Only Ca(OH) _2_ (positive control)	2.5 ± 1.04	2.33 ± 1.03	5.17 ± 1.83	3.5 ± 1.87
3	Ca(OH) _2_ combined with propolis for 7 days	8.6 ± 1.86	8.83 ± 1.72	9.17 ± 3.31	9.16 ± 1.72
4	Ca(OH) _2_ combined with propolis for 14 days	12.83 ± 2.13	12.50 ± 2.34	10.5 ± 2.42	11.67 ± 2.33

Abbreviations: bFGF, basic fibroblast growth factor; BMP4, bone morphogenetic protein 4; Ca(OH) _2_ , calcium hydroxide; SD, standard deviation.

Data normality was examined using the Shapiro–Wilk's examination, showing that each parameter group's data had a normal distribution. The analysis was then followed by a homogeneity test using the Levene's test, which showed that the data had homogeneity with *p* > 0.05. A one-way ANOVA test was performed to determine the differences between the groups in each parameter and showed that each respective parameter group data had significant differences. We further examined the data with least significant difference (LSD) post hoc multiple comparisons to specify the considerable differences between each respective group of bFGF, beta-catenin, CD105, and BMP4.

Based on the LSD post hoc test, all the bFGF groups from the control group to 14 days of propolis Ca(OH) _2_ combination group have significant differences. The same result was also found in the BMP4 group that the LSD post hoc test reported substantial differences between each respective group.

Different results were found in the CD105 group. No significant differences were found between the positive control (A1) group treated with only Ca(OH) _2_ and the control treated with aqua dest. In the beta-catenin group, we found that no significant differences were found between the positive control (A1) group that treated with only Ca(OH) _2_ , the control that treated with aqua dest, the positive control (A1) group with only Ca(OH) _2_ , A2 group that treated with the 7-day combination of propolis and Ca(OH) _2_ , and A3 group treated with the 14-day combination of propolis and Ca(OH) _2_ . Significant results were found between each respective group, which is not stated in the statement earlier.

## Discussion

From the average table stated earlier, we found that in the group that received Ca(OH) _2,_ multiple growth parameters of the proliferation of cells decreased than the parameters in the group that received aqua dest only. This finding resembles the *in vitro* study by Khosropanah et al that investigates the cytotoxicity and proliferation of cells in a different dose of Ca(OH) _2_ and a combination of adjuvant material with Ca(OH) _2_ at different time frames. They found that a higher amount of Ca(OH) _2_ has cytotoxic properties and reduces the proliferation of cells. Adjuvant material that buffers the pH of Ca(OH) _2_ is also beneficial to the survivability of the cells. 12

Studies also found that the utilization of Ca(OH) _2_ has weaknesses besides its actual properties. 13 The relative shortcoming of the density allows bacteria to penetrate tissue and worsen the condition of tissue that already endures the alkalinity effect of Ca(OH) _2_ . Both infection and toxicity effects form necrotic pulp tissue which also poses a problem due to the prolonged inflammatory effect that follows the phenomenon. 14 Prolonged inflammation also will widen the tissue damage and intercept wound healing. 15

We examined beta-catenin, one of the growth parameters, and we will discuss the first one. Beta-catenin regulates several genes in different physiological processes and is helped by runt-related transcription factor 2 (Runx2), a transcription factor that allows osteoblasts and odontoblasts to form tissue lining. 16 Beta-catenin decreased at a level, although not statistically significant, as in the report above. Statistically, beta-catenin did not respond significantly when compared with Ca(OH) _2_ and when combined with propolis, except for the more extended administration period.

This phenomenon can happen because beta-catenin is complex and intertwines with another pathway that may be hard to nail down. 17 Beta-catenin is also known to have dual antagonist activating factors. For example, proinflammatory and anti-inflammatory modulators can upregulate or downregulate beta-catenin release. 18 Ca(OH) _2_ admission also influences the microenvironment with calcium ion release over time, which may affect calcium ion levels in the intracellular environment. However, concentration will diminish bit by bit with increased time. 19 Ca(OH) _2_ effect and microenvironment change may affect the beta-catenin release and its upstream pathway. 20

Calcium ions released by Ca(OH) _2_ paste can shift the Wnt signaling pathway from the canonical pathway that generates beta-catenin to the noncanonical pathway. Noncanonical Wnt pathway can inhibit beta-catenin release in multiple pathways. 21 First was the inhibition by activation of the CaMKII-TAK1-Nemo-like kinase pathway. 22 The second was by nuclear factor of activated T cells (NFAT)-mediated transcriptional regulation. 23 Calcium ions can also be detected by calcium-sensing receptors (CaSR) that examine extracellular calcium levels over time. 24 Activated CaSR will promote protein kinase C alpha to phosphorylate the beta-catenin that proteasome will degrade. The inhibition effect of beta-catenin will be diminished when calcium ion release decreases with time. 25 26 A study shows that Ca(OH) _2_ with water paste will release 70% of its calcium ion within 7 days. 23 In statistics, enhancement with propolis occurred slowly and did not seem to contribute significantly within 7 days either.

Diminishing calcium ion release also explains why the CD105 reduction in the Ca(OH) _2_ -only group was not significantly different from that in the aqua dest-only group within 7 days. Yet, the angiogenesis mechanism's inhibition was still visible in the mean value result of CD105. 8 27 CD105 generally presents itself in mesenchymal stem cells for forming reparative dentin in the dental pulp and also functions to fix tissue damage and angiogenesis. 28 29

Combination with propolis significantly increased CD105 level, which indicates angiogenesis increase. CD105 is a coreceptor of transforming growth factor (TGF)-beta and is essential in wound healing. 8 CAPE is known to help accelerate the inflammation process and release growth factor mediators, including TGF-beta, in the final phase of wound inflammation. 30 This research collected tissue samples 7 and 14 days after induction and considered them to be already entering the resolution phase. The higher values of CD105 at 14 days also proved this rationale.

bFGF is a secretory signaling protein that belongs to the FGF family and is expressed in various tissues. Molecules of BFGF are also secreted by cells indirectly with a translocation between membrane cells that involves cell proliferation, cell differentiation, cell migration, angiogenesis, and cell survival in the physiological or pathological state. bFGF can also act by binding to tyrosine kinase FGF receptor 2 (FGFR1-4) in membrane cells and is essential in teeth development. In this research, bFGF was increased following treatments with a combination of propolis to the maximum to stimulate angiogenesis in the dental pulp. 10 31

Propolis contains CAPE with antioxidant properties that control the reactive oxygen produced during the acute phase of inflammation. CAPE also has an anti-inflammatory mechanism by inhibiting the cyclooxygenase mechanism and the production of proinflammatory cytokines. CAPE also stimulates the production of anti-inflammatory cytokines that help shift immune cells toward regenerative mechanisms. 30 Those mechanisms are essential for tissue regeneration and differentiation, as demonstrated by the increasing levels of bFGF 14 days after propolis admission.

BMP4 is a member of the TGF-beta superfamily, and its mechanism is related to TGF-beta, an anti-inflammatory cytokine whose release aligns with CAPE's anti-inflammatory properties. 8 30 The BMP pathway is essential in mesenchymal and dental epithelial interaction. 32 BMP pathways can regulate intracellular and extracellular factors so that dynamic balances between intracellular and extracellular factors can be achieved. BMP4 expression is the first BMP that humans can identify in the interaction between dental epithelium and mesenchyme in hopes it can initiate dental pulp proliferation and differentiation. 33 34

Clinical issues are expected outcomes of dental pulp disorders, which caries, trauma, or other forms of irreparable damage can cause. DPSCs, biomaterials, and growth factors have been the center of attention in the field of tissue engineering as of late. The dental pulp is continuously undergoing angiogenesis, the process of blood vessel creation. Angiopoietin-2 (ANG2) and its receptor tyrosine kinase TIE2, as well as hypoxia-inducible factor 1-alpha, are crucial components in the process of angiogenesis. 35 36 Understanding the molecular pathways aids in guiding regenerative treatments, even when the study design may not explicitly demonstrate these changes in tissues. To investigate functional pulp regeneration, preclinical trials frequently employ animal models and need to be investigated.

Based on the post hoc test, it is concluded that both bFGF and BMP4 are better than the other growth parameters. Both bFGF and BMP4 displayed significant differences in all the groups from the control to the propolis admission group. That parameter is helpful for further study in evaluating tissue regeneration, proliferation, and differentiation of pulp tissue with better sensitivity than another growth parameter we include in this study. This study also revealed that a combination of propolis shows the formation of dental reparative that goes progressively and significantly. However, we would like to remind the reader about the limitations that may arise because of the design of this study, like the confounding bias that may influence the model result estimates.

## Limitations of This Study

This study did not compare for density; it only mixed Ca(OH) _2_ paste with propolis so that the consistency was similar to that of the paste pulp capping and was homogeneous. It did not test the density of Ca(OH) _2_ paste with the paste that had been formed because it had to be measured so that the density was more objective.

## Conclusion

Propolis mixed with Ca(OH) _2_ in the long term (14 days) significantly improves the growth parameters of bFGF and BMP4, which can be used statistically to better appraise growth from reparative dentin with propolis.
